# Perspectives of female medical faculty in Ethiopia on a leadership fellowship program

**DOI:** 10.5116/ijme.5985.f644

**Published:** 2017-09-01

**Authors:** Elizabeth Kvach, Bethlehem Yesehak, Hiwot Abebaw, James Conniff, Heidi Busse, Cynthia Haq

**Affiliations:** 1 Department of Family Medicine, Denver Health/ University of Colorado-Denver, USA; 2 School of Medicine, Addis Ababa University, College of Health Sciences, Ethiopia; 3 Department of Family Medicine, University of Minnesota-Duluth, USA; 4 School of Human Ecology, University of Wisconsin-Madison, USA; 5 Department of Family Medicine and Community Health, University of Wisconsin-Madison, USA

**Keywords:** Women, gender equity, leadership training, Ethiopia, academic medicine

## Abstract

**Objectives:**

This study aims to evaluate a leadership fellowship program through perspectives of Ethiopian women medical faculty participants.

**Methods:**

An intensive two-week leadership development fellowship was designed for women faculty from Ethiopian medical schools and conducted from 2011-2015 at the University of Wisconsin-School of Medicine and Public Health in Madison, Wisconsin. Nine Ethiopian women working in early- or mid-level academic positions were selected. Semi-structured interviews were conducted with the fellows. Transcripts were reviewed through qualitative analysis to assess the perceived impact of the training on their careers. Three male academic leaders were interviewed to solicit feedback on the program.

**Results:**

Eight of 9 fellows were interviewed. Themes describing the benefits of the fellowship included: increased awareness of gender inequities; enhanced motivation for career advancement; increased personal confidence; and improved leadership skills. Fellows provided suggestions for future training and scaling up efforts to promote gender equity. Male leaders described the benefits of men promoting gender equity within academic health centers.

**Conclusions:**

This paper provides evidence that targeted brief training programs can enhance women’s motivation and skills to become effective leaders in academic medicine in Ethiopia. Promoting gender equity in academic medicine is an important strategy to address health workforce shortages and to provide professional role models for female students in the health professions.

## Introduction

Only approximately one in ten Ethiopian girls will accomplish her dream of advancing to post-secondary education; fewer numbers will complete graduate or professional training.^1^ The women who are able to achieve these milestones often must overcome significant obstacles related to their gender, including cultural expectations, gender discrimination, an internalized sense of inferiority, lack of financial resources, and gender-based violence (GBV).^2^ In light of these obstacles, it is not surprising that few women rise to become faculty and into leadership positions.

Factors contributing to Ethiopian women’s low participation in leadership are complex. Approximately 90% of Ethiopian girls enrol in primary school, but this drops to less than 50% in secondary school, and 20-30% at the undergraduate and postgraduate levels. Even fewer women graduate.^1^ The lifetime prevalence of GBV against Ethiopian women is approximately 75%.^3^^-^^4^ Studies addressing the challenges faced by women in Ethiopian post-secondary educational institutions have identified that gender discrimination and GBV, including sexual harassment, are common occurrences.^5^^-^^7^ This leads to decreased rates of matriculation, increased rates of depression with suicidal thoughts, and contributes to poor academic performance and higher attrition among female students and faculty.^8^^-^^9^ Meanwhile, a national survey conducted in 2011 found that just over 50% of women reported being formally employed in the prior 12 months.  About one-third of these women, in contrast to 9% of men, were not paid for their work. Ethiopian women perform mostly agricultural work, and are less likely than men to engage in professional, technical, or managerial fields.^2^^,^^10^

Gender inequality, as defined by the United Nations Development Program, refers to the differences in achievements between men and women within a nation. This difference is reflected through the gender inequality index (GII), which compares educational attainment, economic status, reproductive health, and political representation between men and women.  The higher the GII value, the greater the difference in achievements between women and men. In general, European countries have the lowest GII (Slovenia is 2.1%), while nations in central Asia and sub-Saharan Africa have the highest GII. The most recent GII of Ethiopia was 54.7%.^11^ The paucity of women occupying major institutional and political leadership positions is both a cause of and a result of gender inequality in Ethiopia.

At Addis Ababa University’s School of Medicine (AAU-SoM), the oldest and largest medical school in Ethiopia, few Ethiopian women serve in academic leadership roles, despite being an important and growing percentage of students and the health professional workforce. As of 2015, only 20% of faculty were female. Among these, over three-fourths held lower ranks of lecturer or assistant professor; at the time of this writing only one female faculty was a full professor. 

Increasing female leadership, particularly in health care, is one critical step towards reducing gender inequality in society.  A few studies have found that women leaders are more likely to focus on issues that directly improve the lives of women, such as access to health care, clean water supply, improved education and income generation.^12^ Women in leadership roles serve as role models for female students, particularly in academic institutions.

In 2010, AAU-SoM was selected as the lead institution for a consortium of Ethiopian and US medical schools for a five-year Medical Education Partnership Initiative (MEPI) grant awarded by the US government.  The aims of MEPI in Ethiopia were to strengthen the capacity of Ethiopia’s medical education system by improving the quality of undergraduate medical school curricula, expanding opportunities for faculty development, and promoting medical research.^13^ Due to the aforementioned level of institutional inequity between men and women, all MEPI-Ethiopia partner institutions (Addis Ababa, Hawassa, and Haramaya Universities, and the Defense Medical College) were expected to promote gender equity as a condition of the grant award. This strategic objective was achieved by school administrators and MEPI consortium members who selected promotion of women into leadership positions as a priority. This objective was achieved through a series of coordinated activities, including providing an intensive fellowship to cultivate leadership skills among female Ethiopian faculty.

Nine female faculty from AAU, Hawassa and Haramaya Universities were selected by their respective administrations to complete fellowship trainings between 2011-2015. The same fellowship content was conducted in three separate sessions during this period for new fellows who were recruited. The fellowships were limited to two weeks to minimize fellows’ time away from personal and professional responsibilities. The fellowships were conducted at the University of Wisconsin-Madison (UW) in Wisconsin, US, with funding provided by MEPI.^14^

UW faculty leaders conducted a pre-fellowship needs assessment of fellows in order to guide development of curricular objectives and content. By the end of the fellowship, participants were expected to achieve the following objectives:  1) Describe concepts of gender equity and health equity research; 2) plan a project to promote gender equity in their home institution; 3) prepare a professional development plan, including leadership, research and teaching goals, potential challenges, and strategies to achieve their goals; 4) present a summary of their proposals to UW faculty leaders; 5) network and collaborate with external partners; and 5) serve as academic leaders and champions for gender equity in their home institutions and in  Ethiopia.^14^

Curricular content was adapted from a women’s leadership program developed by the Association of American Medical Colleges (AAMC).^15^ See Table 1 for a detailed curriculum outline. After completion of fellowships, UW faculty traveled to Ethiopia at least once per year for in-person meetings, and fellows were encouraged to maintain contact with their UW mentors for at least one year following the fellowship to report on progress of their independent projects.

The purpose of this study was to evaluate the perspectives of Ethiopian women medical faculty who completed the fellowship, and to summarize male leaders’ perspectives regarding how to promote women’s leadership within academic medical institutions in Ethiopia. Additionally, we report the professional positions of fellows before and after the fellowship.

## Methods

### Study design and participants

Semi-structured interviews^16^were conducted individually with eight of nine female fellows between February and August 2015 at AAU-CHS. One fellow was completing psychiatry training abroad and was unable to participate. Topics covered during the interviews included, but were not limited to: career progress and satisfaction; future career goals; barriers to ideal careers; perceived effects of gender on career; perceived strengths of fellowship; suggestions for changes to the fellowship; activities and barriers related to gender equity undertaken since the fellowship; and overall assessment of the fellowship (see Appendix I). Interview questions were developed by the authors. Fellows were asked to provide their professional positions and roles before and after the fellowship.

**Table 1:** Gender equity fellowship curriculum outline

Fellowship curriculum outline
• Share personal stories and establish professional goals
• Read and discuss theories of leadership
• Meet with female and male leaders to discuss common challenges faced by women in academic leadership roles
• Review the content of programs designed to train women for academic leadership
• Review UW policies/programs designed to prevent and address sexual harassment and to promote a gender-friendly climate
• Develop gender equity policies relevant to the Ethiopian context
• Outline the components of mentoring programs to support the professional development of faculty and students
• Meet with colleagues of similar specialties to explore future collaboration
• Participate in sessions on health equity research in a pre-existing Health Equity Leadership Institute offered by the UW Collaborative Center for Health Equity
• Build supportive relationships and network with UW colleagues.

Semi-structured interviews were also conducted with three male faculty leaders, two of whom were the principal investigators of the MEPI grant at AAU-SoM and Hawassa University. Questions included: perceptions of pre-existing gender inequities and gender equity efforts; assessment of the fellowships; and future institutional goals related to gender equity (see Appendix I).

Interviews were conducted by three trained interviewers. Interviews were conducted in English and audio recorded then transcribed. Personal identifiers were removed from the interview transcripts to maintain confidentiality. The transcriptions were manually coded and themes and sub-themes were extracted into deductive categories that aligned with the study questions as described by Emerson et al (2005).^17^ Frequency of themes and sub-themes were then identified and summarized. One reviewer performed the initial coding and theme extraction which was then reviewed by all co-authors to ensure accordance on the themes identified.

Ethical considerations of the study included maintaining anonymity of the participants given the sensitive nature of the topics discussed. The Addis Ababa University-College of Health Sciences Institutional Review Board granted this program evaluation exemption from formal approval and considered it not human subjects research.  Informed consent was obtained from each fellow prior to conducting the interviews. Responses were aggregated anonymously and direct quotations were de-identified of any information that could be associated with individual participants.

## Results

Fellows ranged in age from mid-20s to 40s, and represented the departments of Obstetrics/Gynecology, Radiology, Neurosurgery, Psychiatry, Physiology, Microbiology, Pharmacology, and Pediatrics. Major interview themes and sub-themes are summarized in Table 2.

### Career progress, satisfaction and future goals

All eight fellows interviewed reported a positive outlook and overall satisfaction with their careers to date. Fellows were working in public and private institutions after completion of the fellowships. Professional positions held by fellows pre- and post-fellowship are outlined in Table 3. Of note, one fellow commented that multiple factors influenced her career choices before and after the fellowship.

Specific factors that contributed to fellows’ job satisfaction included enjoying teaching and mentoring students, and a sense of deep professional commitment to provide service to the people of Ethiopia. One fellow commented that her long-term involvement in departmental leadership over many years afforded her the ability to recognize her own personal contributions. At its inception, her department was heavily dependent on external expatriate support to function.  Over time, it became self-sustainable to train graduates to serve the country.

Many fellows felt the guidance they received from the fellowship influenced their career ambitions, stating,

*“After I took that leadership training, I was motivated and I have different skills. Before the fellowship and after the fellowship, I was not the same.” *(Fellow 1, female)

Fellows’ future career goals included: academic advancement; expansion of clinical skills training; participation in research; developing greater work-life balance; increasing gender equity activities at the university level; engaging in advocacy, national policy development and practice guidelines; and volunteering in the community outside of their academic positions. Four fellows indicated their desire to eventually become full professors and two others planned to seek leadership positions within their departments. All fellows indicated interest in greater involvement in clinical or translational research that could lead to advancements in clinical care for patients in Ethiopia. Of note, a few fellows had difficulty conceptualizing career goals ten years into the future, which they attributed to uncertainty of institutional leadership and resources, and the shifting nature of government policies.

**Table 2:** Summary of themes and sub-themes of outcomes of a leadership fellowship for female Ethiopian health science faculty

Theme	Sub-Theme	Direct Quotes
Career progress	• Overall satisfaction • Future goals	“I can say that I am very much happy. I am satisfied with my profession and with the things that I have accomplished so far.” (Fellow 6, female) “After I took that leadership training, I was motivated and I have different skills. Before the fellowship and after the fellowship, I was not the same.” (Fellow 3, female)
Barriers to desired career	• Lack of resources • Excess responsibilities • Scarcity of advancement opportunities for women • Effect of gender	“I enjoy teaching, although it’s not very easy to do research because of so many problems, I’m trying now to get involved in student’s research.” (Fellow 3, female) “…I do feel because when you are a female even if you have a job that you earn something, you contribute to the economy of the family, but still when you go back home you are a woman.” (Fellow 4, female) “I don’t feel like [gender] has been to [my] advantage. I feel we had to work twice as hard to prove ourselves.” (Fellow 8, female)
Advantages/strengths of fellowship	• Development of professional skills, including positive attitude and motivation • Leadership training, increased personal confidence • Time for reflection and study, including work/life balance • Consciousness of gender issues, locally and globally • Mentorship	“It has helped me see the importance of working with others, listening to each other, the importance of being part of a team, the importance of listening more rather than speaking more. I have met a lot of people and have been inspired. This fellowship has helped me a lot.” (Fellow 8, female) “If we work hard on them, and if we are able to change the mentality of the people, we can even improve the health care system and the medical education, even without a lot of resources.” (Fellow 7, female) “I really hope we can see more change in the future. I’m also glad that one of the things that I learned from this fellowship is that even a little change or even a little contribution is good, instead of no change.” (Fellow 7, female) “The women in academic leadership training was a very timely training for me. Because I was the head of department at that time but as you know we don’t have any basic skills, tools and equipment of leadership, except the will to serve. But the training in academic leadership came just a few months before I became a dean. So the training definitely opened my eyes. I met a lot of great people and attended very critical courses which helped me to become a better leader than I was before. Even if it was a short training, it was intense and purposeful. I am sure in the few years I have been a dean, most of the good things that I have done came from the tools that I have acquired from the training. And I am sure it will not stop there, whatever I do in the future the skills I got from the training will be very useful.” (Fellow 5, female) “I want myself to be a holistic person.” (Fellow 8, female) “Another very important area for me personally is work life balance and I think it is also important for so many women as we have many responsibilities in our life. It is important to think about how the institution can be more flexible on this area. It is to some degree a policy issue and we need to work on it.” (Fellow 6, female) “I think the most important part of the workshop is it showed me that it is okay to acknowledge there are gender issues and it is also right to face them.” (Fellow 5, female) “Because you would think that people in that position in developed countries do not have that problem… It doesn’t mean you are developed or underdeveloped, it just means you are a woman.” (Fellow 3, female) “The most useful thing I found is seeing that there is a gender issue in any institution. In the legal, political and social environment we live in. We usually feel like there is no gender issue but there is. And it would be a big lie to say there are no gender issues in Ethiopia, maybe it is better than some developing countries in Africa but we are never spared.” (Fellow 5, female) “I have seen women who have had it all, women who have had their careers, their families, and leadership positions. I’ve seen that it can be balanced. It can be done.” (Fellow 7, female) “In the area of leadership when I was exposed to such kind of training I was able to see that there are things like having a role model and being a role model as well as being mentored and mentoring others which will help us be the best of what we are.” (Fellow 6, female)
Suggestions for change to fellowship curriculum/structure	• Adaptation of curriculum to Ethiopian context • Sustained follow-up	“Another thing I would change would be, if it is possible, to give this training in Ethiopia. We could reach more people, more female faculty.” (Fellow 7, female) “I think the most important thing for the fellowship program is to make it contextualized. In our faculty we now have more young and diverse faculty both socially and academically so contextualizing and diversifying the course will be very much important.” (Fellow 5, female) “Only having such training by itself may not be enough. The components like leadership, mentoring, career development, looking at the problems institutionally…these are big areas and [we need] to become more expert by further training… I suggest some [more] follow-up and support at the level of the institute or the country. We need to form a body having these issues at hand, sharing experience with one other country is not enough…the training is a good beginning but will not help the country as much [without additional follow-up].” (Fellow 6, female)
Gender equity activities undertaken since fellowship completion	• Sessions for female students and residents • Mentoring • Research • Institutional policy and advocacy • National organization	“We are involved in one of the studies that we conducted and meetings that we had also for gender equity. That was really good because a lot of people came from each department. There was a really good talk with different topics about gender equity, and then the paper – the preliminary data was presented, and people were giving very positive feedback.” (Fellow 3, female) “There are many things that influence you, so in addition to this I have a friend who works in gender, who is outside of this university. So she usually invites me to meetings associated with gender, and then we are involved now in the association of women scientists.” (Fellow 3, female)

**Table 3:** Professional positions of fellows before and after the fellowship*

Pre-Fellowship Professional Position	Post-Fellowship Professional Position
• Assistant Professor of Physiology	Assistant Professor of Physiology
• Associate Professor of Microbiology, Head of Microbiology Department	Associate Professor of Microbiology; Head of Institutional Review Board
• Lecturer in Psychiatry • Department Head of Psychiatry	Private practice psychiatry
• Department Head of Obstetrics & Gynecology, Assistant Professor of Obstetrics and Gynecology	Dean of Medical School, then Assistant Professor of Obstetrics and Gynecology
• Assistant Professor of Pharmacology	Assistant Professor of Pharmacology, Department Head of Pharmacology
• Assistant Professor of Pediatrics, Pediatric Endocrinologist	Assistant Professor of Pediatrics, Pediatric Endocrinologist, Private practice
• General practitioner	Lecturer, neurosurgery resident
• General practitioner	Lecturer, radiology resident
• Assistant Professor of Psychiatry	Psychiatry subspecialty fellowship in Canada

*At the time of writing

### Barriers to career goals

When asked about barriers to their career goals, the following major obstacles were mentioned: lack of resources, including administrative support; excessive personal and professional responsibilities resulting in feeling overloaded; a perceived scarcity of advancement opportunities for women; and lack of institutional protections. All fellows expressed frustration with limited opportunities for grants and institutional resources to fund research. For clinical fellows, the lack of resources in the Ethiopian medical system to provide adequate medical care for patients was noted as a particular hindrance.  Fellows who advanced into leadership roles were often asked to assume new responsibilities in addition to their prior roles, leaving insufficient time for pre-existing duties and to pursue academic interests. Fellows in these situations felt increasing burn-out, and two stepped down from leadership positions as a result.  For some fellows, the low number of specialists in their fields compounded this problem, placing pressure on them to provide essential patient care, medical education, and departmental leadership simultaneously. One fellow reported being denied an academic position at a public university by a male administrator, despite being the only fellowship-trained sub-specialist in her field in the entire country. Several women mentioned barriers related to gender including: a lack of female heads of department in any of the basic sciences; having to perform their own secretarial work even when they were in leadership positions; and the double burden of responsibilities at home as mothers and wives in addition to their professional duties. One fellow complained,

*“…I do feel because when you are a female even if you have a job that you earn something, you contribute to the economy of the family, but still when you go back home you are a woman.” *(Fellow 4, female)

Another fellow was informed while she was on maternity leave that another person had been appointed to replace her. However, using skills she gained in the fellowship, she advocated for herself, asserting,

*“What is my sin? Is it being a female, giving birth and leaving for some time? They had no… answer and returned me to my position.” *(Fellow 1, female)

Most fellows did not feel that their gender had been a disadvantage or an advantage to their careers. However, the fellow who experienced discrimination during her maternity leave clearly identified her gender and childbirth as the primary cause, and the two youngest fellows, who recently completed medical internships, noted negative treatment attributable to their gender. One reported treatment from male classmates that she later recognized as sexual harassment, stating that it made an “uncomfortable environment” for her as a woman. (Fellow 7, female)

### Advantages and strengths of the fellowship

All fellows interviewed would recommend the fellowship to their colleagues. Five major sub-themes of personal and professional benefits from the fellowship were identified (see Table 2). The first sub-theme is the development of pragmatic professional skills, including introduction to grant and proposal writing; mentoring younger women; and engaging in quality improvement projects. In addition, sharing of institutional resources from the UW Office of Diversity such as the sexual harassment policy was mentioned as a useful tool that was being adapted to their home institutions.

Fellows also identified development of a positive attitude and motivation to influence future changes as a valuable acquired skill. One fellow illustrated this by stating,

*“I really hope we can see more change in the future.  I’m also glad that one of the things that I learned from this fellowship is that even a little change or even a little contribution is good, instead of no change.” *(Fellow 7, female)

A second sub-theme was the expansion of leadership skills, including increased self-confidence and ambition for career advancement. One of the first fellows declared,

*“Because of the Wisconsin fellowship, I became so bold!” *(Fellow 2, female)

Fellows mentioned specific benefits of leadership training such as learning to listen more carefully to others, how to work in teams, exploring concepts of leadership, and sustaining persistence in the face of challenges. Regarding the aforementioned fellow who faced difficulty during her maternity leave, the experience inspired her to apply to a master’s course in health leadership to pursue more senior positions. She said,

*“Unless females hold a higher influential position, it is very difficult to change the root problem of gender inequities in Ethiopia, particularly at higher institutions.” *(Fellow 1, female)

Another fellow gained understanding of how to say ‘no’ and step down from roles when overburdened with too much work in order to be able to focus on work related to her priorities. She reflected,

*“…You need to know what you want, and to be more assertive in what you really want.” *(Fellow 3, female)

A third sub-theme was having structured time away from personal and professional responsibilities to reflect with other women on their careers, discuss work-life balance, and envision goals for the future.  One fellow noted that it was helpful to have space to discuss past negative experiences. She had experienced a difficult situation within her department that made her not want to pursue a leadership position in the future.  The fellowship helped her deconstruct this experience and reconsider a future leadership role.

The fourth sub-theme was the development of a consciousness of gender issues as a starting point to motivate future actions to reduce gender inequities in their home institutions. Many fellows noted that their conversations with women from the US raised their awareness that there are transnational cross-cutting issues that can affect all women because of their gender. This led to hope for the possibility of change in Ethiopia. One fellow observed,

*“Because you would think that people in that position in developed countries do not have that problem…  It doesn’t mean you are developed or underdeveloped, it just means you are a woman.” *(Fellow 3, female)

Two fellows were not able to articulate direct benefits of the fellowship. However, during the course of the interview, they described distinct changes in their motivations and the ways in which they interacted with female students. They both pursued a research study to investigate the extent of GBV and discrimination among female students and faculty (results in process). In addition, they shared examples of their heightened sensitivity toward the needs of female students, and of reaching out to mentor female students. One told a story of a female graduate student who was struggling with her coursework. The fellow invited the student to her office and discovered she was a single mother raising four children. Subsequently the fellow offered this student more support and encouragement. That student is now progressing successfully in a PhD program.

The final subtheme raised was the importance of mentorship, both in terms of being mentored and becoming a mentor to more junior women faculty and students. All fellows discovered that mentoring could be strengthened in their home institutions, and could become an important source of support for the advancement of women in academic medicine. As one fellow reported,

*“In the area of leadership, when I was exposed to such training, I was able to see that there are things like having a role model and being a role model as well as being mentored and mentoring others which will help us be the best of what we are.” *(Fellow 6, female)

### Recommendations for changes to the fellowship

Fellows provided recommendations to improve future fellowships if they were to be offered. Fellows suggested that the curriculum content should be made more specific to the Ethiopian context, and requested extra time for reading, personal reflection, and discussion. They proposed devoting more time to development and critique of grant writing skills and career plans with individualized discussion and feedback. One fellow suggested the fellowship should be 3-4 weeks in duration. Most fellows desired longer-term support after completing the fellowship.  This support could include mentoring, ongoing training and skills-development, helping to recruit younger women, and assisting their efforts to promote change at institutional and national levels. One fellow noted that the fellowship was only a first step, and that much more work needed to be done at local and national levels to effect meaningful changes in women’s roles and leadership in Ethiopia,

“Only having such training by itself may not be enough. The components like leadership, mentoring, career development, looking at the problems institutionally… these are big areas and [we need] to become experts by further training. I suggest some [more] follow-up and support at the level of the institution or the country. We need to form a body having these issues at hand, sharing experiences with one other country is not enough… the training is a good beginning, but will not help the country as much [without additional follow-up].” (Fellow 6, female)

Regarding the structure of the fellowship, several fellows felt it should be offered in Ethiopia in order to be able to reach more women. They recommended that more than one woman at a time should be selected from an institution in order to achieve a critical mass to effect change, and that Ethiopians should lead future fellowships for other Ethiopian women. Several fellows suggested involving men for some parts of the program.

### Gender equity activities undertaken since fellowship completion

Multiple activities were undertaken by fellows after completing the fellowship to promote gender equity at their respective institutions. Three fellows reported holding sessions for female undergraduates and residents regarding GBV, leadership, stress management, study skills, and balancing roles as a woman. One fellow advocated for female residents who were forced to take examinations during their maternity leave, and had worked to create more family-friendly options for residents to return part-time and to extend the duration of their training. Four fellows provided individual mentoring of female students and felt that their students opened up more readily to female faculty.

Two fellows, in collaboration with international partners, completed a gender climate survey to assess prevalence of GBV and associated socio-demographic characteristics of female students and faculty (results in process). This data helped formulate a gender equity action plan that was presented to university administrators as a roadmap for future change. The administration has been working to institute this plan in collaboration with two other fellows who participated in the committee.  Fellows developed a code of conduct to prevent sexual harassment that was approved by the faculty senate.  After the code was approved, fellows worked with the AAU gender office to provide trainings on the new policy to AAU College of Health Sciences students and staff. Six fellows were involved in the development of a gender office and its subsequent activities. This included the development of school materials to empower “underprivileged” female students to avoid exploitative, “sugar daddy” relationships with older men, relationships that could put them at risk of GBV, HIV, and pregnancy. These same fellows were involved in helping to organize and participate in a series of college-wide meetings for women and men on gender climate. These meetings provided a forum for students to air grievances and to raise awareness about gender equity.

Two fellows became involved in women’s issues at the national level. One is actively involved in the Ethiopian Association of Women Scientists, and another is working with another female physician to launch an Ethiopian Medical Women’s Association.

One fellow reported she had not undertaken any gender equity-related activities since the fellowship because she left the institution with which she had originally been affiliated.

### Male leaders

All three male leaders interviewed acknowledged that the low percentage of women in the student body and faculty was a major problem exacerbated by entrenched cultural and religious views, a high prevalence of gender-based violence, and a lack of institutional support such as paid maternity leave. One declared,

*“Without gender equity, no society will achieve its maximum level. You’re leaving 50% of the community behind. Without gender equity, no development will take place. It is not just about advancing the status of women, but about uplifting all of society.” *(Leader 1, male)

All male leaders expressed positive opinions about the fellowships in terms of their impact on participants and their institutions.  They recommended that more women should be trained to bring about major institutional changes; that men should be involved; there should be greater institutional support for gender equity efforts; fellows should lead training for additional women; and graduates should work as a team to relieve individual burdens and sustain progress. These suggestions aligned with feedback from the fellows.  Male leaders reflected that men’s participation was critical to change institutional cultures to promote gender equity. However, this study did not establish whether these sentiments were representative of and shared by the broader male faculty population, an area for potential future research.

### Future actions

All fellows identified goals to promote gender equity in their institutions. These included: strengthen gender offices with enhanced resources and trained staff; establish a formal mentorship program for students and faculty; periodically repeat gender climate surveys to assess progress; conduct regular meetings with female students to elicit concerns; establish a counseling program for female students; develop formal curricula to introduce gender issues at the undergraduate level; and reduce disparities between increasing numbers of female medical students and the small percentage of female residents and faculty. One fellow articulated the importance of addressing this issue of female medical student attrition and lack of promotion to faculty positions by stating:

*“I believe this shouldn’t continue in the coming generation.  We have to put a stop to it. A good proportion of females should go into residency programs and faculty and leadership positions. This should be seriously addressed in whatever possible way.” *(Fellow 5, female)

Male leaders proposed the following future actions: institutional and community awareness-raising campaigns; inclusion of men in gender equity trainings; continued fellowships with larger numbers of women; providing incentives to recruit and retain women through affirmative actions, housing and other benefits; changing institutional policies to promote gender equity specifically regarding GBV and harassment; and setting aside at least one day per year to “celebrate gender” and discuss gender issues.

## Discussion

This study demonstrates that a brief, targeted intervention can enhance female faculty members’ motivation and skills to promote gender equity in Ethiopian medical schools. To our knowledge this is the first program to design, offer and evaluate leadership training for women in academic medicine in sub-Saharan Africa.

Ethiopian women have historically been less likely to enter higher education and to serve as leaders in academic medicine due to cultural norms, limited educational opportunities, harassment, and discrimination.^5^^-^^8^ Many Ethiopian women have previously considered leadership roles as unattainable since the majority of these roles have been held by men and there are few female role models. Leadership roles are often considered unattractive to women and men alike due to perceptions that these roles are conflict-laden with high costs and few rewards. However, these norms are changing in Ethiopia, as they are in most of the world.

Each of the fellows had already demonstrated remarkable leadership qualities by overcoming numerous challenges to complete medical training in a country with relatively few female physicians or faculty members. They reported the fellowships provided both individual and institutional benefits, including time to focus and reflect on gender discrimination, strategies to promote gender equity, and opportunities to enhance their leadership skills. Fellows expressed deep commitment to improving health care in Ethiopia and satisfaction with their careers to-date. They also shared frustrations, experiences of discrimination, and episodes of harassment. They described double burdens of home and professional responsibilities that contributed to fears of becoming overwhelmed and burnt out. Yet despite these challenges, fellows appreciated opportunities to enhance their skills with faculty from an outside institution in a safe environment. The program provided fellows with role models, motivation, self-confidence, and new perspectives and tools. Since completing the fellowships, each of the participants has sustained progress in their careers and contributed to initiatives that have benefitted themselves and others.

There is little to no literature regarding career advancement of women in academic medicine in Ethiopia or sub-Saharan Africa. One study in the United States evaluated three career development programs for women in academic medicine that were offered from 1988-2010, including the AAMC program upon which the Ethiopia fellowship curriculum was based. Past female participants (n=845) were surveyed and 95% of respondents who attended a course early in their career (e.g., as an assistant professor) reviewed it positively and reported gains in skills as a result of the course, the highest ranked of which were interpersonal skills, leadership, networking, negotiation, and planning for promotion.^18^ The first three skills (interpersonal, leadership, and networking) are similar to those reported by fellows in our study. This would seem to indicate that Western curricula for women’s leadership can be adapted to non-Western settings with beneficial outcomes. However, adaptation should occur through consultation with local partners to ensure relevance to local cultures and contexts. For example, policies and criteria for promotion in academic rank may differ between countries. Thus, guidance on planning for promotion should be adjusted accordingly.

As a result of the fellowship, participants were motivated and able to envision new policies to change their institutional cultures. They conducted meetings with students, staff, and faculty to raise awareness and to propose ambitious gender equity action plans.  They conducted a college-wide survey of female faculty and trainees to assess the gender climate. They launched new courses on gender and health for all students and designed new programs to prepare students with leadership skills.

Promotion of positive changes in institutional gender climate is likely important for retention of female students and faculty within all educational institutions. The rates of matriculation for female students in the AAU-CHS have been steadily increasing, but the attrition rate for female students is higher than that for male students.^14^ Evidence on mentoring in academic medicine suggests that women perceive greater difficulty finding a mentor, but if they do have one they report a positive impact on personal development, career guidance and choice, and productivity.^19^ In societies such as Ethiopia where traditional gender roles are more rigidly defined, it may be difficult for female students and junior faculty to find a willing or competent mentor. And yet, increasing the number of female faculty role models and mentors through future leadership development is an important step to recruit and retain women in medical careers and academic medicine.

Male leaders agreed on gender equity as a priority of the MEPI initiative and devoted resources to support the fellowships and follow-up activities. They selected the female fellows and encouraged and follow-up actions. They welcomed women into leadership roles and co-sponsored many new initiatives.  One principal investigator of the MEPI grant shared his perspective, “Without gender equity, no society will achieve its maximum level. It is just like clapping with one hand.”  Based on experience from the US to promote gender equity and careers of women in academic medicine, involvement of administrative leaders is crucial to enact and sustain significant long-term institutional changes.^20^ Therefore, changes are more likely to be successful if institutional leaders are engaged and invested in the outcomes.

While we are encouraged by these outcomes, we are aware of the limitations of this study. This was a short-term fellowship with intensive resources devoted to a relatively small number of female participants.  The fellows were purposively selected based on previously demonstrated leadership characteristics. Therefore, it is likely that all fellows would have continued to progress in their careers whether or not they participated in the fellowship. Due to the short time period of follow-up, we cannot predict whether the fellowship will have sustained impacts on the professional career paths of individuals or on their institutions. Finally, given our lack of ability to control for a multitude of factors beyond this intervention that could have influenced the fellows’ accomplishments, we are unable to determine whether the fellowships definitely contributed to positive career outcomes.

## Conclusions

The leadership fellowship program for Ethiopian women in academic medicine described in this study is a small but significant step for a country which is working to increase the number of women in higher education and leadership positions. Nevertheless, we are encouraged by the findings from this short-term intervention to raise awareness, empower participants, and begin to change institutional cultures to promote gender and health equity. Some students and faculty are celebrating and benefitting from this intervention. We are hopeful that these changes will be sustained and expand over time.

We consider these findings as encouraging indicators that times are changing. Faculty in Ethiopia are improving opportunities for women in medicine and in society. We advocate for more widespread programs to promote gender equity to be designed, scaled-up, evaluated and refined for greater impact and the benefit of future generation students and faculty in medical education.

### Acknowledgments

We are grateful for the courage and contributions of Drs. Mahlet Gebremariam, Yimtubezinash Woldeamanuel, Tewabech Zewde, Miheret Tamirat, Fiker Taddesse, Rihanna Abdurhman, and Bereket Fantahun. This work was made possible by the leadership of Drs. Miliard Derbew, Girma Tefera, Dawit Desalegn, Abebe Bekele, Belayhun Temesgen and the support of MEPI Ethiopia staff including Rahel Bisetegne, Netsanet Animut, Deseret Mulatu and Habtamu Melese. Many UW faculty, staff and students generously contributed their time to host the fellows including: Alex Adams, Fiona Adams, Lori DiPrete Brown, Sabrina Butteris, Susan Davidson, Sarah Esmond, Alida Evans, Ann Evensen, Rosa Garner, Valerie Gilchrist, Elizabeth Jacobs, Judith Leavitt, Heather Lukolyo, Patrick McBride, Elizabeth Petty, Luis Pinero, Laurel Rice, Christie Seibert, Karin Silet, Betsy Teigland, Brenda Gonzalez and Ellen Wald.

The funding for this work came from the Health Resources and Service Administration (HRSA) under the terms of the grant for the Medical Education Partnership Initiative (MEPI), Ethiopia (T84HA21124).

### Conflict of Interest

The authors declare that they have no conflict of interest.

## Supplementary material

Supplementary file 1 Appendix I. Semi-structured interview instrument for male and female fellows
