# Supplementary material for: Perspectives of female medical faculty in Ethiopia on a leadership fellowship program
Source: Int J Med Educ. 2017 Sep 1;8:314–23. doi: 10.5116/ijme.5985.f644 (PMC5694698; doi:10.5116/ijme.5985.f644)
Supplement: Supplementary file 1 — Appendix I. Semi-structured interview instrument for male and female fellows [file ijme-8-314-S1.pdf]

Appendix I

A. Semi-Structured Interview Instrument for Female Fellows

- What is your age and current professional position? What was your professional position prior to the fellowship?
- How do you feel about the course of your career so far?
- What would you like to be different, if anything?
- What things would you like to accomplish that you haven't yet?
- Where do you see yourself in 5-10 years time?
- Do you feel like the leadership training fellowship helped you personally or with your career? In what ways?
- What work have you done at your home institution to help promote gender equity?
- Do you feel your gender has been to your advantage or disadvantage to your career as a physician in Ethiopia? If so, then why?
- Looking back on the fellowship, what did you find the most useful?
- If you were to offer the fellowship for others, what would you change?
- Would you recommend the fellowship to female colleagues?

B. Semi-Structured Interview Instrument for Male Leaders

- What is your age and current professional position?
- Do you feel gender equity is important in Ethiopia? Why or why not?
- What are the major issues related to gender equity in your medical school?
- What are the major obstacles to achieving gender equity?
- What is your vision for gender equity in Ethiopia? How would things be different?
- What are the most important steps to achieve gender equity in Ethiopia?
- Would you recommend continuation or expansion of the gender equity fellowship? If so, how could it be improved?
